# The Kinetics of Plasmacytoid Dendritic Cell Accumulation in the Pancreas of the NOD Mouse during the Early Phases of Insulitis

**DOI:** 10.1371/journal.pone.0055071

**Published:** 2013-01-25

**Authors:** Jojanneke M. C. Welzen-Coppens, Cornelia G. van Helden-Meeuwsen, Pieter J. M. Leenen, Hemmo A. Drexhage, Marjan A. Versnel

**Affiliations:** Department of Immunology, Erasmus MC University Medical Center, Rotterdam, The Netherlands; Wayne State University, United States of America

## Abstract

In non-obese diabetic (NOD) mice that spontaneously develop autoimmune diabetes, plasmacytoid dendritic cells (pDCs) have a diabetes-promoting role through IFN-α production on one hand, while a diabetes-inhibiting role through indoleamine 2,3-dioxygenase (IDO) production on the other. Little is known about the kinetics and phenotype of pDCs in the NOD pancreas during the development of autoimmune diabetes. While para/peri-insular accumulation of conventional dendritic cells (cDCs) could be observed from 4 weeks of age onwards in NOD mice, pDCs only started to accumulate around the islets of Langerhans from 10 weeks onwards, which is concomitant with the influx of lymphocytes. NOD pancreatic pDCs showed a tolerogenic phenotype as assessed by their high expression of IDO and non-detectable levels of IFN-α and MxA. Furthermore, expression of the pDC-attracting chemokines CXCL10 and CXCL12 was significantly increased in the NOD pancreas at 10 weeks and the circulating pDC numbers were increased at 4 and 10 weeks. Our data suggest that a simultaneous accumulation of IDO^+^ pDCs and lymphocytes in the pancreas in 10 weeks old NOD mice, which may reflect both an immunogenic influx of T cells as well as a tolerogenic attempt to control these immunogenic T cells.

## Introduction

The nonobese diabetic (NOD) mouse model is a widely used animal model of type 1 diabetes (T1D) [1] and characteristically develops lymphocyte accumulations around the islets of Langerhans from the age of 10 weeks onwards [2], [3]. These cellular accumulations subsequently progress to infiltration of the islets and finally destruction of the insulin-producing beta cells. Prior to the lymphocyte accumulation, an increased influx of conventional dendritic cells (cDCs) into the NOD pancreas from 4 weeks onwards can be observed and these cDCs concentrate in and around the islets [4], [5]. Previously, we showed in a depletion study using clodronate-loaded liposomes, that these early accumulating mDCs are essential for the recruitment of lymphocytes into the NOD pancreas [6]. A study by Saxena et al. [7] using conditional knock-out mice confirmed these observations and showed that an early temporal depletion of mDCs totally abrogated the development of insulitis and diabetes in the NOD mouse model.

In addition to mDCs, distinct and vital roles for pDCs have also been described to be important in the development and progression of diabetes [8], [9]. In mice pDCs are characterized as CD11b^−^CD11c^low^B220^+^PDCA-1^+^Siglec-H^+^ cells, and these cells express the chemokine receptors CCR5, −7, CXCR3 and −4 [9]. pDCs are classically known as important mediators of antiviral immunity through their ability to produce large quantities of type I interferons (IFN) upon viral infection and stimulation of the appropriate toll-like receptors (TLRs) [8]. While virally activated pDCs act as immunogenic cells, resting or alternatively activated pDCs have been described to have tolerogenic activity [10], [11]. Interestingly, an aberrant chronic pDC activation and secretion of type I IFN has been found in systemic autoimmune diseases such as systemic lupus erythematosus (SLE) and Sjögren’s Syndrome, and it is thought to play an important role in the pathogenesis of these systemic autoimmune disorders [12]–[15].

In the development of autoimmune diabetes in NOD mice, both a pathogenic and tolerogenic role for pDCs has been described. pDCs were found to be pathogenic and contribute to disease development through IFN-α production on one hand [7], [16], [17], while depletion of pDCs on the other led to an acceleration of insulitis and a loss of local indoleamine 2,3-dioxygenase (IDO) [7], [18]. It has been suggested that the aggressiveness of the NOD insulitis is controlled by local tolerogenic pDCs and production of IDO, which could induce deletion of self-reactive cells by depleting the cells of tryptophan or could stimulate the development of regulatory T cells through the production of kynurenine products [19]. pDCs could also act in a tolerogenic manner through interaction between the programmed death ligand 1 (PD-L1) on pDCs and PD-1 on T cells, which delivers inhibitory signals to T cells [20]. However, whether this pathway plays a role in the tolerogenic function of pDCs in NOD autoimmune diabetes has not yet been addressed. In fact, little is known about the kinetics of pDC accumulation in the NOD pancreas during the spontaneous development of the autoimmune insulitis process.

Here we investigated the presence and localization of pDCs in the NOD pancreas during the early phases of insulitis (4 and 10 weeks). We analyzed their expression of IDO and PD-L1 as well as local IFN production, both by protein expression and by induction of the IFN-inducible gene myxovirus (influenza) resistance A (MxA). In addition, we studied the pancreatic expression of chemokines that are important for the attraction of pDCs from the circulation and enumerated pDCs in the circulation of the NOD mouse in the early phases of the insulitis process.

## Materials and Methods

### Animals

C57BL/6 and NOD/shiLTj female mice were obtained from Charles River Laboratories (Maastricht, The Netherlands) and NOR/LTj mice from the Jackson Laboratory (Bar Harbor, ME, USA). Mice of 4, 10 and 20 weeks of age were used and housed under specific pathogen-free conditions.

### Ethics Statement

Sampling of the mice was approved by the Animal Experiments committee of the Erasmus MC (Dierexperimentencommissie (DEC), which is the ethical committee installed and officially recognized as required by the Dutch Law on Experimental Animals, the Dutch analogue for the IACUC). The approval number is: DEC#2334, dated June 15, 2011. The study was conducted in compliance with all relevant Dutch laws and in agreement with international and scientific standards and guidelines.

### Immunohistochemistry

Cryostat sections (6 µm) of pancreases of C57BL/6, NOR and NOD mice were prepared and fixed with cold methanol and acetone. Slides were incubated with rat-anti-Siglec-H (both eBiosciences, San Diego, CA, USA) or rat-anti-IDO (Biolegend, San Diego, CA, USA) followed by rabbit-anti-rat-PO (DAKO, Glostrup, Denmark). Subsequently, slides were incubated with Nickel-DAB and counterstained with nuclear fast red (both Sigma Aldrich, St. Louis, MO, USA), followed by mounting in Entallan (Merck, Darmstadt, Germany). Insulitis was evaluated by the analysis of at least 50 islets according the following scale: 0, no infiltrating cells; 1, few infiltrating cells peri-insular; 2, large numbers of infiltrating cells around the islet and 3, large numbers of infiltrating cells in the islet.

### Immunofluorescence

Cryostat sections (6 µm) of pancreases of C57BL/6 and NOD mice of were prepared and fixed with cold methanol and acetone. Slides were incubated with rat-anti-IDO followed by goat-anti-rat-TexasRed (Southern Biotechnology Associates Inc., Birmingham, AL, USA) and rat-anti-Siglec-H-FITC (eBiosciences). Finally, slides were mounted in Vectashield with DAPI (Vector Laboratories Inc., Burlingame, CA, USA).

### Preparation of Cell Suspensions

Pancreases were isolated after a cardiac perfusion and cut into small pieces and digested with collagenase type 1 (1 mg/ml), hyaluronidase (2 mg/ml) (both Sigma Aldrich, St. Louis, MO, USA) and DNAse I (0.3 mg/ml) (Roche Diagnostics, Almere, The Netherlands) for 40 minutes at 37°C. Subsequently, cells were flushed through a 70 µm filter and washed. Blood was collected in EDTA tubes after a cardiac punction. Erythrocytes were lysed with NH_4_Cl buffer and cells washed with PBS. All cells were resuspended in PBS containing 0.1% BSA followed by flow cytometric staining.

### Flow Cytometry

Single-cell suspensions from pancreas were labeled with CD45 beads (Miltenyi, Leiden, The Netherlands) and CD45^+^ cells were sorted using AutoMACS (Miltenyi) and labeled with mAbs. Single-cell suspensions from blood were labeled with mAbs. Antibodies used were B220-Pacific Blue, CCR5-PE, CCR7-PE-Cy7, CXCR3-PE, CXCR4-APC, CD11b-APC-Cy7, CD80-PE-Cy5, CD86-APC, PDCA-1-FITC and PD-L1-PE (all eBiosciences, San Diego, CA, USA). Afterwards cells were washed and resuspended in 0.1% BSA/0.5% paraformaldehyde, followed by analysis on a FACS Canto HTSII (Becton Dickinson) flow cytometer and FACS Diva and Flowjo software.

### Protein Expression Determination

Pancreas lysates of C57BL/6, NOR and NOD mice were prepared by homogenization of the pancreas in ice-cold PBS supplemented with protease inhibitor cocktail (Life Technologies, Paisley, UK). The lysates were sonicated twice for 30 seconds and centrifuged at 1000 g at 4°C for 10 minutes. The supernatant was collected and stored at −80°C. The protein concentration in the pancreas lysates was determined using the Bradford method (Bio-rad Laboratories GmbH, München, Germany).

The pancreas lysates were tested for CXCL10 protein expression using a cytometric bead array according to the manufacturer’s protocol (eBiosciences). Briefly, a mixture of beads coated with antibodies against CXCL10 was incubated with the lysate or standard mixture. A biotin-conjugated second antibody mixture was added followed by streptavidin-PE. Samples were analyzed using a FACS Canto HTSII (Becton Dickinson) and FlowCytomix Pro Software (eBiosciences). CXCL9, −11 and −12 (R&D Systems, Minneapolis, MN, USA) and IFN-α (PBL Interferon source) protein expression were determined using ELISA kits according to the manufacturers protocol.

### Statistical Analysis

Data were analyzed by Mann-Whitney U test for unpaired data. All analyses were carried out using SPSS software (SPSS Inc., Chicago, IL, USA) and considered statistically significant if p<0.05.

## Results

### pDCs Accumulate Around the Islets in the NOD Pancreas Concomitant with Lymphocytes, but Later than mDCs

The localization of mDCs and pDCs in the pancreas of NOD, C57BL/6, and nonobese diabetic resistant (NOR) mice of 4 and 10 weeks was studied by immunohistochemistry. The NOR mouse is a NOD-related MHC-syngeneic strain, and often used as a control strain for the NOD mouse model. NOR mice do not develop diabetes and only have a mild lymphocytic peri-insulitis [21].

**Figure 1:**
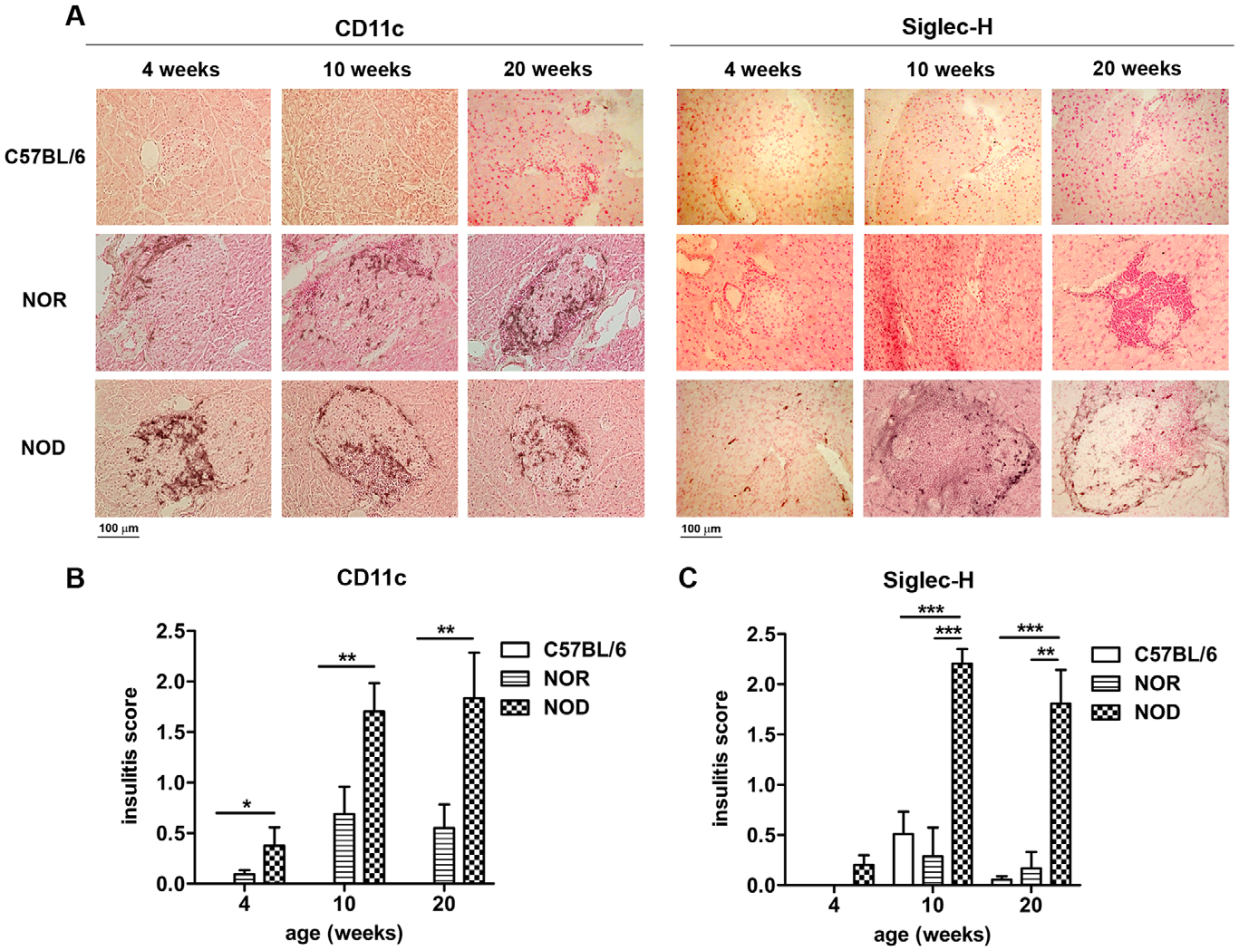
Accumulation of mDCs and pDCs in the NOD pancreas. The localization of mDCs and pDCs in the C57BL/6, NOR and NOD pancreas at the age of 4, 10 and 20 weeks was determined by immunohistochemical detection. Pictures show CD11c and Siglec-H expression in the pancreas of mice from 4, 10 and 20 weeks of age, magnification 200x (A). Bar graphs represent the mean insulitis score of CD11c^+^ (B) and Siglec-H^+^ cells (C) in the pancreas. Data are presented as average+SEM, n = 5 mice, * p<0.04, ** p<0.01, *** p<0.001 as determined by the unpaired Mann-Whitney U test.

From 4 weeks onwards CD11c^+^ mDCs were observed to accumulate around the islets in the NOD pancreas (Fig. 1A and B). The peri-islet accumulation of CD11c^+^ mDCs was also observed in the NOR pancreas, although at reduced numbers. Siglec-H^+^ pDCs were detectable in the exocrine pancreas of NOD mice from 4 weeks onwards, but significant differences were not detected between NOD and control strains. At 10 weeks the lymphocytic para/peri-insulitis had started in the NOD mouse, at that time mDCs had become more numerous and pDCs also had started to accumulate at the islet edges (Fig. 1A). The number of Siglec-H^+^ pDCs was significantly higher in the NOD pancreas as compared to NOR and C57BL/6 at 10 and 20 weeks (Fig. 1C). In the NOD pancreas the levels of mDCs and pDCs at 20 weeks were not significantly different from the NOD levels at 10 weeks.

**Figure 2:**
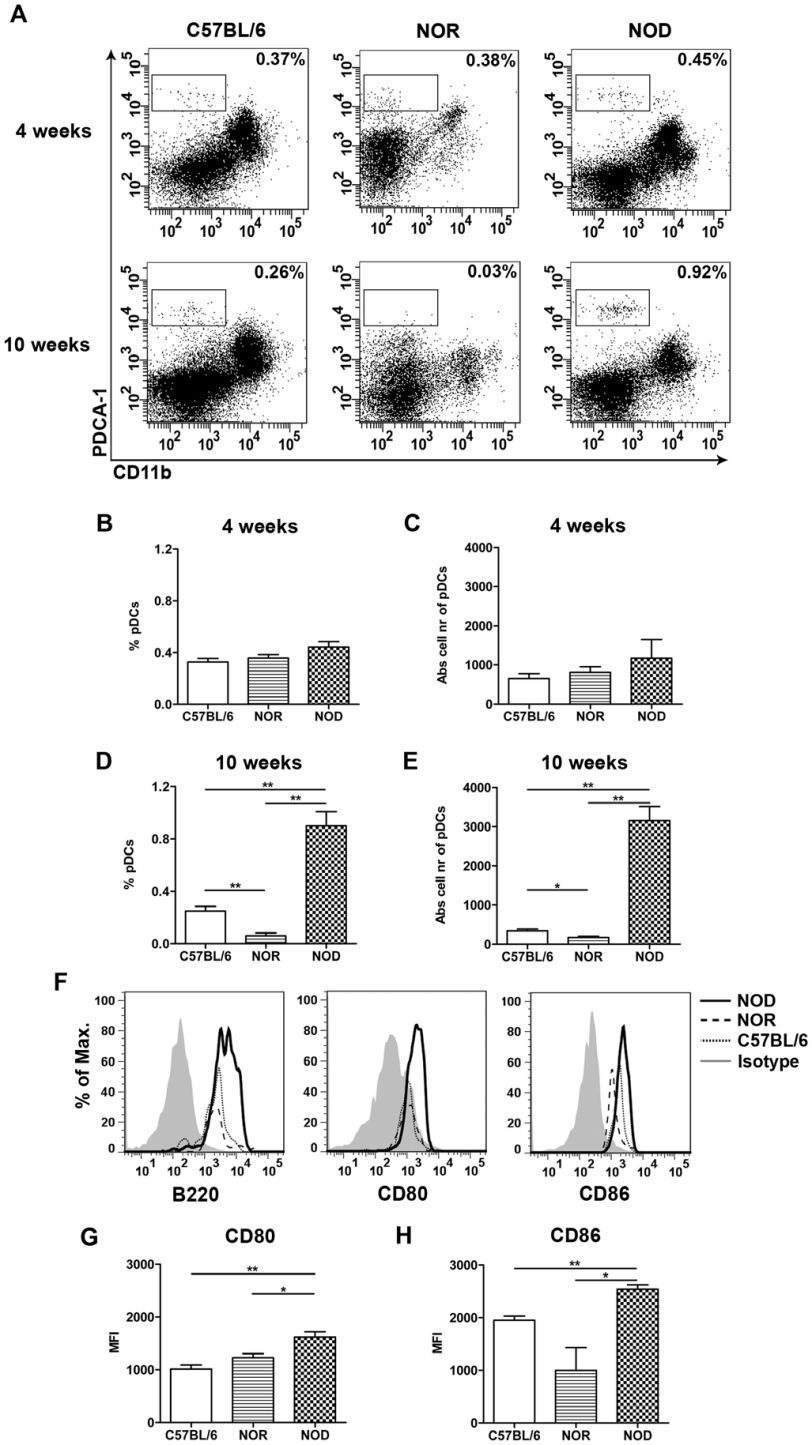
Increased pDC numbers in the NOD pancreas at 10 weeks of age. The presence of pDCs in the C57BL/6, NOR and NOD pancreas at the age of 4 and 10 weeks was determined by flow cytometry. Dot plots show the CD11b and PDCA-1 expression on sorted CD45^+^ cells from the pancreas (A). Bar graphs represent the percentage and the absolute number of CD11b^−^PDCA-1^+^ cells in the pancreas at 4 (B–C) and 10 weeks (D–E). Histograms represent the B220, CD80 and CD86 expression on pDCs (CD11b^−^PDCA-1^+^ cells) in the pancreas of 10 weeks (F). Bar graphs represent the geometric MFI of CD80 (G) and CD86 (H) on pDCs. Data are presented as average+SEM, n = 5–6 mice, *p<0.04, ** p<0.01 as determined by the unpaired Mann-Whitney U test.

In addition to the immunohistochemistry, flow cytometry was performed on sorted CD45^+^ cells from the pancreas of 4 and 10 week old C57BL/6, NOR and NOD mice. As Siglec-H was not detectable after the pancreatic digestion (data not shown), pDCs were identified by PDCA-1 staining. No differences in CD11b^−^PDCA-1^+^ pDCs were observed between the strains at 4 weeks of age (Fig. 2A–C). However, at 10 weeks both the percentage and absolute number of CD11b^−^PDCA-1^+^ pDCs was significantly increased in NOD mice compared to controls (Fig. 2A, D and E). All strains showed similar expression of B220 in the CD11b^−^PDCA-1^+^ pDC population (Fig. 2F), but CD80 and CD86 were significantly increased in NOD mice (Fig. 2F–H), suggesting increased level of activation.

### Enhanced IDO, but Decreased PD-L1 and Absent IFN-α Expression of NOD Pancreatic pDCs

**Figure 3:**
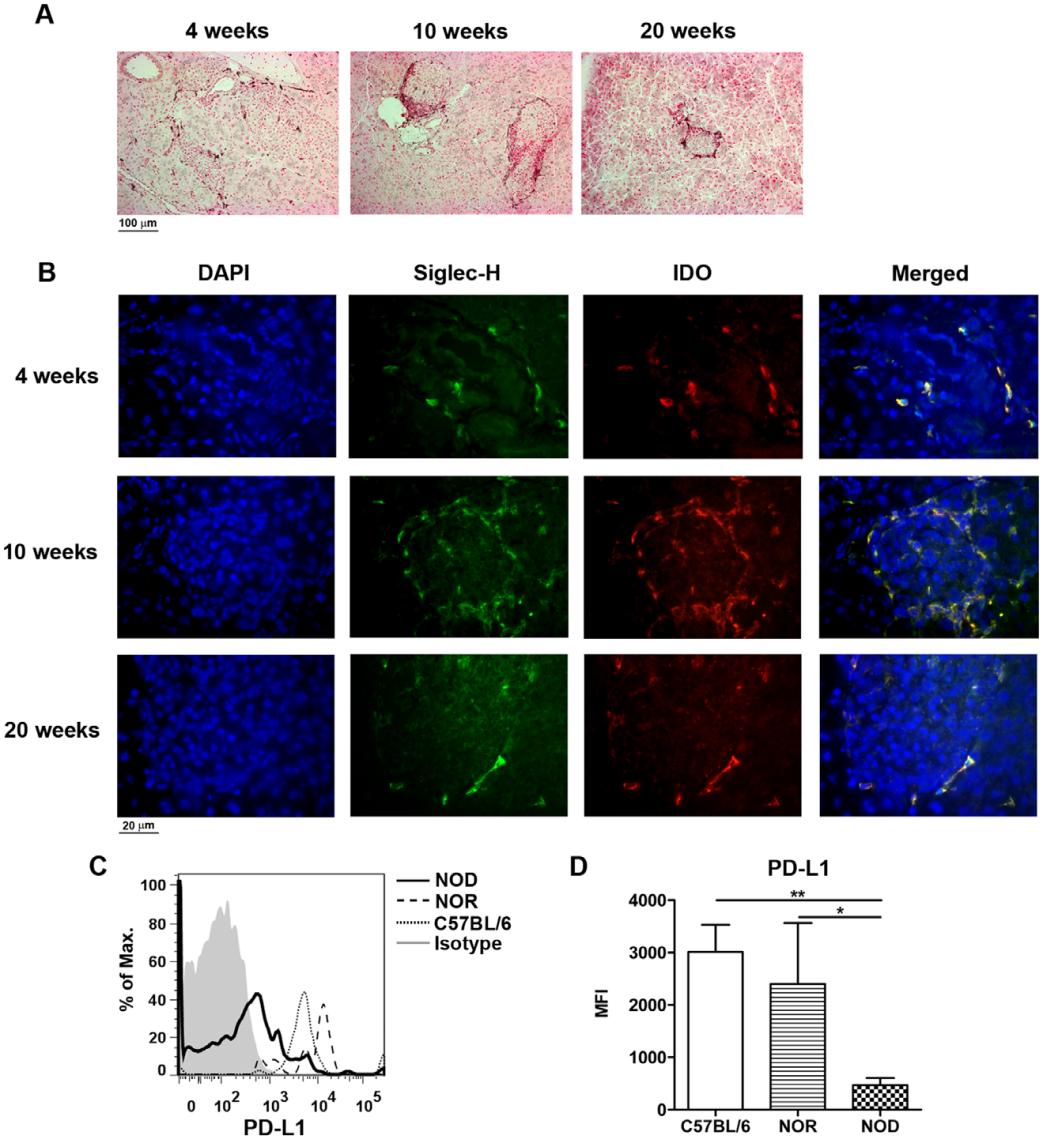
Enhanced IDO and a decreased PD-L1 expression in NOD pancreas pDCs. Pictures show the immunohistochemical detection of IDO in the pancreas of NOD mice at 4, 10 and 20 weeks of age, magnification 200x (A). The pancreas of NOD mice was stained for Siglec-H (green), IDO (red) and DAPI (blue) by immunofluorescence, magnification 400x (B). Histogram represents the PD-L1 expression on pDCs (CD11b^−^PDCA-1^+^ cells) in the pancreas of C57BL/6, NOR and NOD mice of 10 weeks of age (C). Bar graph represents the geometric MFI of PD-L1 on pDCs (D). Data are presented as average+SEM, n = 5 mice, * p<0.04, ** p<0.01 as determined by the unpaired Mann-Whitney U test.

As NOD pancreatic pDCs expressed increased levels of co-stimulatory molecules, immunohistochemical analysis of both tolerogenic and activational markers was performed. In 4 week old NOD mice low numbers of IDO^+^ cells were detected near blood vessels in the exocrine pancreas. From 10 weeks onwards, when pDCs were found to accumulate at the islet edges, IDO^+^ cells were also detected both around and in the islets (Fig. 3A). Immunofluorescent analysis confirmed that IDO^+^ cells were Siglec-H^+^ pDCs (Fig. 3B). In the pancreas of C57BL/6 and NOR mice IDO^+^ cells were not detected (data not shown). In contrast, NOD pancreatic pDCs showed significantly decreased expression of the tolerogenic ligand PD-L1 compared to control strains (Fig. 3C and D). As pDCs are important producers of high amounts of IFN-α upon TLR activation by viruses and immune complexes, IFN-α expression in the pancreas was analyzed. No expression of IFN-α protein and the IFN-induced gene MxA mRNA was detected in the pancreas of all mouse strains at 4, 10 and 20 weeks of age (data not shown).

### High Expression of CXCL10 and CXCL12 in the NOD Pancreas at the Time of pDC Accumulation

**Figure 4:**
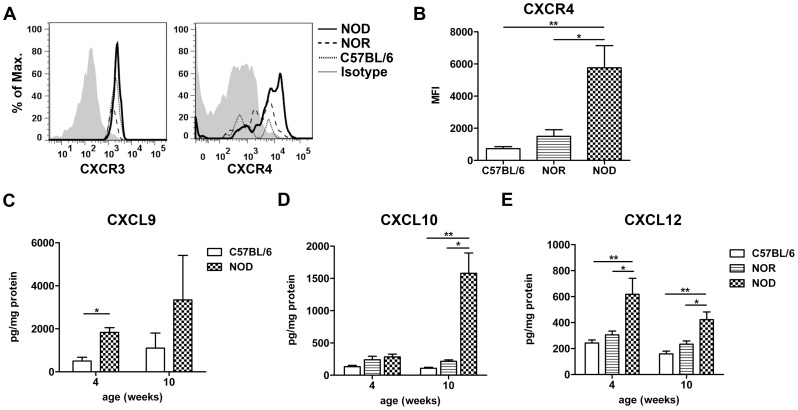
Expression of pDC receptors and chemokines in the pancreas. Histograms represent the CXCR3 and CXCR4 expression on pDCs (CD11b^−^PDCA-1^+^ cells) in the pancreas of 10 weeks of age (A). Bar graph represents the geometric MFI of CXCR4 on pDCs in the pancreas of 10 weeks of age (B). Bar graphs represent the protein level of CXCL9 (C57BL/6 and NOD), CXCL10 and CXCL12 (C57BL/6, NOR and NOD) in pancreas lysates of 4 and 10 weeks (C-E). Data are presented as average+SEM, n = 5–10 mice, * p<0.04, ** p<0.01 as determined by the unpaired Mann-Whitney U test.

To investigate which chemokines and chemokine receptors might be important for the attraction of the pDCs, flow cytometry was performed on pancreatic pDCs. pDCs in the pancreas of all strains expressed similar levels of CXCR3 (Fig. 4A), but NOD pDCs expressed increased levels of CXCR4 (Fig. 4A and B). No expression of CCR5 or CCR7 was detected in all three strains (data not shown).

As pancreatic pDCs expressed CXCR3 and CXCR4, the protein expression of their ligands CXCL9, CXCL10, CXCL11 (for CXCR3) and CXCL12 (for CXCR4) was assessed. CXCL9 and CXCL12 protein levels were significantly increased in the NOD pancreas at 4 weeks of age (Fig. 4C and E), although CXCL9 levels did not reach statistical significance at 10 weeks of age. CXCL10 protein expression levels were only significantly increased in the NOD pancreas at 10 weeks of age (Fig. 4D) and no significant differences were found in the CXCL11 protein expression between the strains (data not shown).

### Increased Numbers of pDCs in the Circulation of NOD Mice

**Figure 5:**
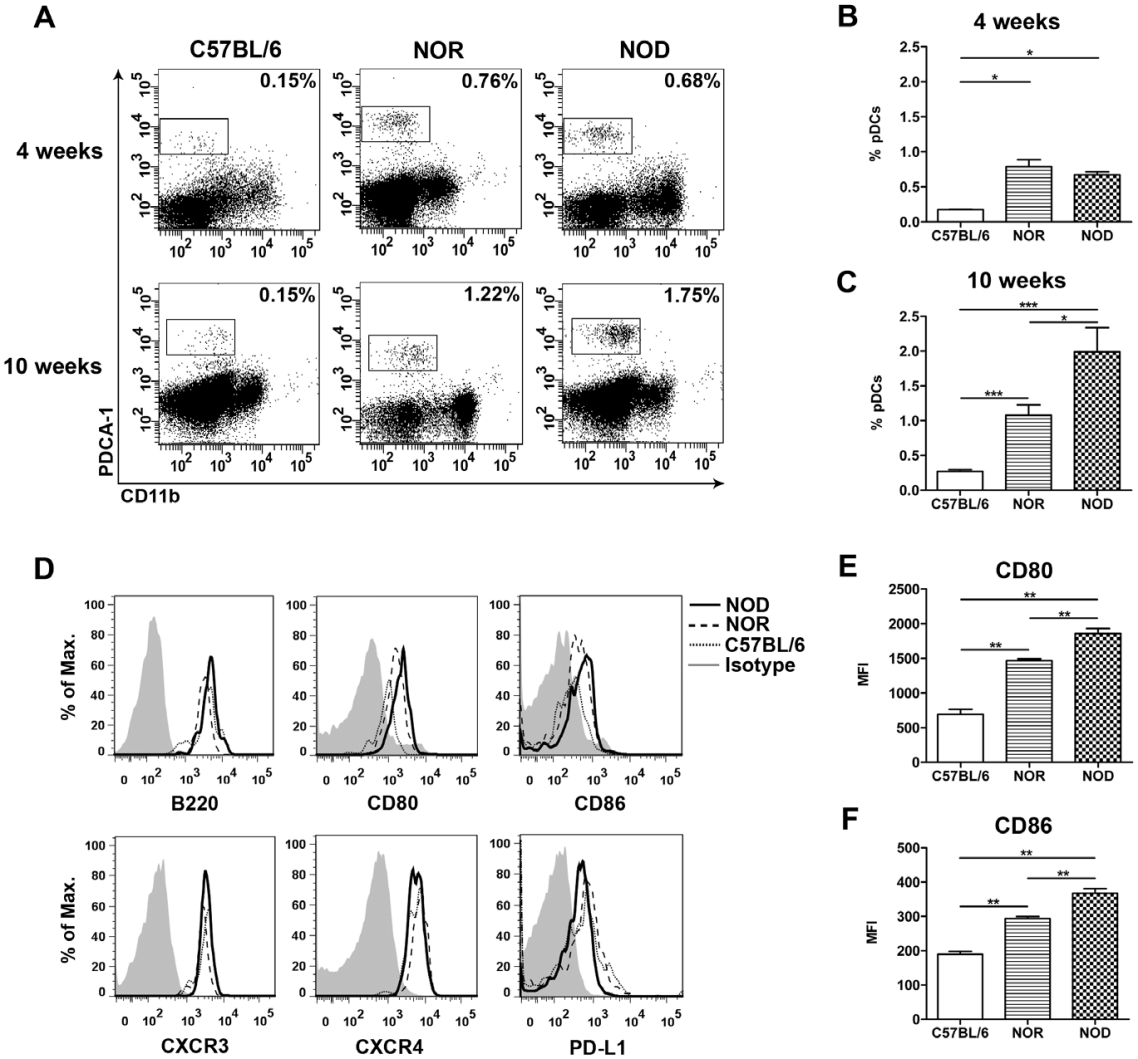
Increased percentage of pDCs in blood of NOR and NOD mice. The presence of pDCs in the blood of C57BL/6, NOD and NOR of 4 and 10 weeks was determined by flow cytometry. Dot plots show the CD11b and PDCA-1 expression (A). Bar graphs represent the percentage of pDCs (CD11b^−^PDCA-1^+^ cells) at 4 weeks (B) and 10 weeks (C). Histograms represent the B220, CD80, CD86, CXCR3, CXCR4 and PD-L1 expression on pDCs in the pancreas of 10 weeks (D). Bar graphs represent the MFI of CD80 (E) and CD86 expression (F) on pDCs at 10 weeks. Data are presented as average+SEM, n = 4–10 mice, * p<0.04, ** p<0.01, *** p<0.001 as determined by the unpaired Mann-Whitney U test.

In peripheral blood, both an increase and reduction in the numbers of circulating pDCs have been described in the various phases of type 1 diabetes (T1D) development in humans [18], [22], [23]. Numbers of pDCs in the blood of C57BL/6, NOR and NOD mice were studied by flow cytometry at 4 and 10 weeks of age. At both ages the percentage of CD11b^−^PDCA-1^+^ pDCs in the blood of NOD and NOR mice was significantly increased as compared to C57BL/6 (Fig. 5A–C). Furthermore, NOD mice showed an increased percentage of pDCs compared to NOR mice at 10 weeks of age (Fig. 5A and C). pDCs were B220^+^, CXCR3^+^, CXCR4^+^ and PD-L1^low^, expressed at similar levels in all 3 strains (Fig. 5D). However, the NOD and NOR pDCs expressed significantly higher levels of the co-stimulatory molecules CD80 and CD86 at 4 (data not shown) and 10 weeks of age compared to C57BL/6 mice (Fig. 5D–F), suggesting increased activation state. Moreover, circulating pDCs in NOD mice also expressed significantly higher levels of CD80 and CD86 compared to NOR mice at 4 (data not shown) and 10 weeks of age (Fig. 5D–F).

## Discussion

This study shows that pDCs with a tolerogenic IDO^+^ profile accumulate at the islet edges in the NOD pancreas at 10 weeks of age. This is at the same time that lymphocytes can be observed to accumulate around the islets [2], [3]. In contrast, the accumulation of immunogenic disease-promoting mDCs occurred earlier from 4 weeks of age onwards. pDCs in the NOD pancreas have a partial tolerogenic profile by expressing IDO, although the PD-L1 expression was lower. Furthermore, no expression of IFN-α and the IFN-induced gene MxA is detected in the NOD pancreas. The pDC increase is accompanied by an increased expression of CXCL10 and −12 in the NOD pancreas.

Saxena et al. [7] described a role for IDO in the down-regulation of the NOD insulitis using an IDO inhibitor, but the actual IDO-producing cells were not identified. Here, we show that pDCs in the NOD pancreas express IDO, identifying a potential local tolerogenic role for these cells. However, pDCs have also been implicated in a disease-promoting role in the NOD mouse: IFN-α^+^ pDCs were increased in the pancreas-draining lymph nodes (pLNs) of 4 week old NOD mice and an antibody to the IFN-α receptor was able to halt the diabetogenic process [24], [25]. In our hands we did not detect significant numbers of pDCs in the NOD pancreas as early as 4 weeks, although we did not analyze pLNs. Furthermore, levels of IFN-α and the interferon-inducible gene MxA were not detectable in both NOD and control mouse strains. Our data would therefore not support an immunogenic function for pancreatic pDCs in the NOD mouse and we favour a tolerogenic role for pancreatic pDCs. Collectively, the literature data and our findings suggest that pDCs in the pLNs early in the process have a different and opposite function (i.e. disease-promoting) as compared to those infiltrating the pancreas at later stages of the disease (disease-inhibiting). While IDO-expressing pDCs were only found in the NOD pancreas, we found a reduced expression level of PD-L1 on these cells compared to pancreatic pDCs in control strains. Interestingly, endocrine cells of inflamed islets of 10 week old pre-diabetic NOD mice also express PD-L1 (but not PD-L2) at 10 weeks of age, and blocking PD-L1 increased insulitis severity and diabetes development in NOD mice [26]. This suggests that PD-L1 may be involved in mediating apoptosis of autoreactive effector T cells in the peri-insulitis, but it remains to be investigated whether this is due to expression of PD-L1 by tolerogenic pDCs or by endocrine cells under immune attack.

A limitation of our study is that the number of pDCs in the pancreas was too low to perform functional studies. Such studies would have strengthened our observations. Another limitation is that the digestion method for the pancreas influenced the expression of Siglec-H. Siglec-H is selectively expressed on pDCs and certain macrophage subsets in the spleen and considered the best marker for pDCs [27], [28]. Since Siglec-H could not be used for the flow cytometric analysis we used PDCA-1 in combination with other phenotypic markers, like CD11b and B220, to distinguish pDCs. PDCA-1, also known as bone marrow stromal antigen-2, is selectively expressed on pDCs, but is also up regulated on other cell types upon type I IFN or IFN-γ stimulation [29]. Despite the reduced specificity of PDCA-1, both immunohistochemistry and flow cytometric analysis suggested an accumulation of pDCs in the NOD pancreas at the time of lymphocyte insulitis, but at a later stage than mDCs.

The mean insulitis score of Siglec-H^+^ pDCs was higher as compared to CD11c^+^ mDCs likely due to the fact that pDCs express low levels of CD11c and the limited sensitivity of this staining.

With regard to the chemokines attracting pDCs, recent studies showed that the expression of CXCL10 alone was not sufficient for pDC recruitment, but that the co-expression of CXCL10 and CXCL12 synergistically was required to induce pDC migration [30], [31]. We observed that the expression of both CXCL10 and CXCL12 was elevated in the NOD pancreas at 10 weeks of age. In addition, the receptor for CXCL12, CXCR4, was significantly increased on pDCs in the NOD pancreas and CXCR3, the receptor for CXCL10, was normally expressed. These findings are supportive of synergistic function of CXCL10 and CXCL12 in the recruitment and retention of pDCs in the NOD pancreas. Moreover, in the pancreas of NOR mice CXCL10 and CXCL12 were not increased and pDC infiltration was not observed. It has been shown that beta cells express CXCL10 during the insulitis in LCMV-infected mice [32] and purified human and rat islet cells have been found to produce CXCL10 upon stimulation with IFN-γ or IL-1β [33]. It is therefore likely that the endocrine cells are the source of these pDC-attracting chemokines, but this needs further investigations.

Several reports have shown reduced pDC numbers in the circulation of patients with recent onset T1D (within 3 months) as well as in patients with long standing T1D (more than 5 years) [22], [23]. Interestingly, a recent study showed an increased frequency of pDCs in the blood of T1D patients at the time of diagnosis, which declined after disease onset [18]. This observation is in line with our data showing an increased pDC number in the blood of NOD mice with active insulitis and points to the putative importance of distinguishing the different phases of disease development in humans and animal models with regard to pDC frequencies in the circulation. Perhaps high numbers of circulating pDCs might be an early sign of an existing (pre-)diabetic insulitis process.

In summary, our present and past studies on the kinetics of the accumulation of the various subsets of DCs in the NOD pancreas during the development of diabetes reveal the following pattern of accumulation:

1. From 4 weeks onwards mDCs start to accumulate in the NOD pancreas. It is our hypothesis that this accumulation of mDCs is (at least in part) due to an aberrant proliferation of local pancreatic precursors for mDCs [34]. Retention of activated and maturing mDC population in the pancreas might be due to an aberrant expression in the pancreas of the lymphoid tissue-specific CCR7 ligands CCL19 and CCL21, as described previously by us [35]. These mDCs are local drivers of the immunogenic effector response towards islet antigens. In addition, a recent study used magnetic resonance imaging (MRI) to visualize local effects of pancreatic-islet inflammation to predict the onset of diabetes in NOD mice [36]. They show that autoimmune diabetes is set at an early age in these mice. In pLNs, mDCs but also IFN-α-producing pDCs are drivers of the immunogenic effector response towards islet antigens.
2. From 10 weeks onwards pDCs and lymphocytes accumulate in the NOD pancreas, possibly attracted by CXCL10 and CXCL12 expression. The infiltrating pDCs express IDO and are meant to dampen the insulitis development in an attempt to halt the insulitis process.
